# Classroom management problems faced by education administrators in distance education during the pandemic period

**DOI:** 10.3389/fpsyg.2022.962559

**Published:** 2022-12-23

**Authors:** Özlem Ece, Mert Bastas

**Affiliations:** ^1^Department of Educational Administration and Supervision, Near East University, Nicosia, Cyprus; ^2^Department of Social Sciences Teaching, Near East University, Nicosia, Cyprus

**Keywords:** COVID-19, education, distance education, teachers, management

## Abstract

Distance education contributes to the development of teamwork skills that may be needed in business life and builds effective collaborative communication skills that allow individuals to learn on their own. Students are able to take online classes, interact with their teachers and become a member of interactive groups. During the pandemic, countries faced different constraints that changed the general perception of education. The use of distance education programs, especially during the recent global COVID-19 epidemic, has had a positive or negative impact on students and had similar impacts on teachers as well. The education sector has also been affected by the various measures taken by countries to minimize the risk of transmission during the pandemic period. In this period, the traditional understanding of education has been interrupted and teachers carried out their education and training activities in technological environments. Distance education has also brought some difficulties as well as provided certain advantages to teachers. Quantitative analysis was preferred as the research method to describe the difficulties teachers faced when implementing open and distance education in Turkey. Within the scope of this study, problems of distance education faced by educators in classroom management are tried to be examined during the pandemic period.

## Introduction

The coronavirus has affected all areas of life and disrupted the traditional understanding of education. Education at all levels has become uncertain due to the mass closures of schools and universities (Sahi et al., 2020). In this process, Turkey took a break from face-to-face education and started distance education to maintain the sustainability of education.

In this process, starting from March 16, 2020, schools in Turkey were suspended until April 30, 2020. In this context, it has been decided to continue education with Open and Distance Education Applications (EBA) with three television channels at the primary and secondary education levels. Due to the ongoing effect of the pandemic, it was announced that the school holiday period was extended until May 31, 2020, and April 29, 2020. It was then decided to continue education online and to review grades for students who completed the first semester (Anadolu Ajansı, 2020).

The main purpose of this research is to examine the difficulties faced by teachers while applying open and distance education regarding the coronavirus (COVID-19) pandemic in Turkey and to evaluate the impact of the pandemic on classroom management, together with generating suggestions for future actions. The main question of the study is, “What kind of difficulties do teachers face when implementing open and distance education in Turkey during the Coronavirus (COVID-19) Epidemic? (Şimşek, 2020).” In this regard, answers to the following questions were sought:

- What kind of difficulties do teachers encounter in classroom management in the distance education application of the Ministry of National Education?
- Which applications are used for open and distance education in the education system?
- What kind of practices can be done to increase the effectiveness of open and distance education applications?

Within the framework of this research, the reflections of the pandemic on education of Turkey and the problems encountered by teachers in classroom management in distance education applied in primary education between March 23, 2021, and April 7, 2021, were investigated based on the primary data on the impact of the coronavirus. The main purpose of the study is to make an overall assessment by identifying the situation and to offer suggestions for future applications for open and distance education. While open and distance education practices continue with the effects of the coronavirus pandemic, the data to be obtained from this study will be of great importance, as it is not yet known when the crisis will end. The results, evaluations, and suggestions presented in this study are important because they contribute to future applications for open and distance education.

The research problem and sub-problems questions are as follows.

1. What are the teachers’ methods of preventing undesirable student behaviors in the classroom?
2. To what extent do teachers encounter undesirable student behaviors while managing classrooms in distance education applications?
3. What preventive strategies do teachers use to address undesirable student behaviors in classroom management in distance education applications?
4. What can be done by teachers to eliminate undesirable technical problems in terms of classroom management in distance education applications?
5. Are there any significant difference among teachers according to their age, gender, or occupational branch while dealing with technical problems of distance education?

In the modern world, which is called the age of communication and information, radical changes called “digitalization” are experienced in people’s lives. According to Friedmann (2006), the instant access of digital technology to everything in our “flattening world” takes people’s living standards to another dimension. In addition, it is imperative to adapt to this rapidly changing and developing age. In many areas of daily life, it is unthinkable that digital technologies do not cover the field of education. Internet connections must be good, where instant feedback from students can be achieved and can be used as an alternative to face-to-face classes (Basilaia et al., 2020). It has been observed that technological materials and digital environments are becoming increasingly prevalent in educational processes. While the concept of distance education is defined by the Turkish Language Association as “a form of education made from a certain center using various communication tools without the student and the teacher face to face,” Schlosser and Simonson (2006) describe distance education as “a combination of teachers and students in different places”.

Distance education institutions have increased the number of distance education programs over the Internet with the benefits offered by the development of information and communication technologies for the changing education and training needs of society. Emerging problems lead to failures in distance education and create some negative prejudices against distance learning. For distance education applications over the Internet to be successful, these applications should be designed to meet the needs and the knowledge and skills that students will gain through face-to-face online learning should be provided (Bilgiç and Tüzün, 2015). So methods to eliminate these kind of communication barriers, by increasing the ability of Internet access, student-to-student interactions, student-to-instructor interactions, student-to-content interactions, and student/instructor motivations are needed to be arranged to make the distance education program more effective (Dabaj and Yetkin, 2011).

In Turkey, due to the coronavirus epidemic, between March 23, 2020, and April 3, 2020, the Ministry of National Education held its lessons in primary, secondary, and high schools on 3 TV channels belonging to EBA and TRT, within the scope of distance education. Ten different courses for primary and secondary schools and 22 different courses for high school are conducted in the form of distance education and with television support. It is noteworthy that the courses offered generally consist of basic courses (mathematics, physics, biology, chemistry, Turkish, etc.) and that other courses (music, physical education, visual arts, painting, etc.) are not included in the application. At the same time, it is seen that the applications for students who need special education are insufficient in terms of quantity and quality.

As in every field, problems will inevitably arise in the field of distance education. These problems undoubtedly affect the success of the distance education field. The introduction of technological means into the educational system with which educational actors interact leads to the emergence of various phenomena of psychological nature: resistance to new technologies and activities, a change in communication style, confidence in interacting with technology, neuropsychic tension, and stress (Lukina et al., 2022). On the other hand, the way to success in distance education passes through solving these problems. It is clear that distance education is irreversible. During the research, the problems related to distance education over the Internet can be grouped under three main headings as “management/organization,” “student,” and “teacher” by scanning the relevant literature and examining the distance education practices in Turkey and abroad. Many research exists in the literature demonstrates the issues and drawbacks of distance education over the Internet and shows that the problems continue to increase in almost all countries. These problems can be listed as follows (Erguney, 2017):

1. Problems that arise in terms of management and organization.
2. Problems faced by students.
3. Problems experienced by teachers.

These problems that may arise with distance education should be taken into account by distance education institutions in all aspects and precautions should be taken in advance.

## Materials and methods

In this section, the exploratory model, population and sample, data collection tool, and data analysis are explained.

### Research model

Quantitative analysis was preferred as the research method. Descriptive analysis was done as a part of this quantitative research to explain the numbers generated after survey results. This method focuses on fresh data collection in accordance with the problem from a large population and analysis of the data is done for description (Rahi, 2017). The variables associated with the study include inappropriate student management behaviors in classroom management in distance education practices and also include teachers’ views on strategies and measures used to prevent undesirable behaviors. Teacher gender, education level, and work experience were selected as independent research variables.

Within the scope of the study, open and distance education applications conducted by the Ministry of National Education between March 21, 2021, and April 23, 2021 as well as distance education data provided between March 23, 2021, and April 07, 2021, are evaluated.

### Population and sample

The study include 100 teachers selected by a disproportionate sample. Disproportionate stratified sampling is a stratified sampling procedure in which the number of elements sampled from each stratum is not proportional to their representation in the total population. Population elements are not given an equal chance to be included in the sample. With disproportionate sampling, the strata selected are not selected pro-rata to their size in the wider population (Fox et al., 2009). The target audience of the research is teachers working in Nicosia schools. The sample of the research consists of 100 teachers working in 10 different schools determined according to the socio-economic level of Nicosia. Sampling participation has been voluntary.

### Reliability analysis

At this stage of the study, the statements about students’ undesirable behaviors, strategies to prevent undesirable behaviors, and intervention methods for undesirable behavior, which are preferred as data collection tools, consist of 35 items in total. When the reliability coefficient of the scales of students’ undesirable behaviors, prevention strategy, and undesirable behaviors intervention methods was examined; these values were determined as 0.877, 0.888, and 0.707, respectively. This value shows that the scales are reliable and that there is no obstacle to its use in the analysis.

### Data collection tools

The questionnaire developed by Sezgin and Duran (2010) was preferred as the data collection tool in the research. The questionnaire consists of four parts. Researchers selected a sample of 100 people to calculate the validity and reliability of the scale and performed factor analysis for the construct validity of the scales and calculated internal consistency coefficients for their reliability (Sezgin and Duran, 2010). Principal component analysis was preferred as the factorization method in factor analysis.

Students’ Undesirable Behaviors Scale (SUBS) consists of 15 questions about the strategies teachers use to prevent undesirable behaviors. The scale has a single-factor structure. The factor loads of the scores in the scale vary between 0.37 and 0.69. The total variance explained in the scale was found to be 28.13%, and the internal consistency coefficient was 0.81. In this study, factor loads were re-calculated and found to be vary between 0.37 and 0.69. The total variance explained was found to be 28.13%, and the internal consistency coefficient was found to be 0.87.

Strategies for Preventing Unwanted Behaviors Scale (SPUBS) consists of 10 questions about the strategies teachers use to prevent undesirable behaviors. The scale has a single-factor structure. The factor loads of the scores in the scale vary between 0.42 and 0.72. The total variance explained in the scale was found to be 35.10%, and the internal consistency coefficient was 0.77. 0.888.

Intervention Methods for Undesirable Behaviors Scale (IMUBS) consists of 10 questions about the strategies teachers use to prevent undesirable behaviors. The scale has a single-factor structure. The factor loads of the scores in the scale vary between 0.41 and 0.62. The total variance explained in the scale was found to be 24.06%, and the internal consistency coefficient was 0.65. 0.707.

### Data collection

The Likert technique assumes that the person understands himself better than the others, and in this technique, the person is expected to give complete and undistorted information about himself. However, it may not always be correct for an individual to provide objective information about their personal thoughts. To prevent this from happening, participants were informed beforehand that their personalities and personal information has been hidden, which led to more candid answers. An online questionnaire has been prepared and sent to school teachers for them to fill out. The participants were allowed to complete the questionnaire at their own pace.

### Data analysis

The analysis of the data was made using IBM SPSS Statistics 26 software. In order to evaluate the reliability of a Likert-type scale, Cronbach’s Alpha Coefficient was used. Data analysis was carried out in two stages. In the first stage, inaccurate or missing values, multiple changes, and outliers were checked in the data transferred to the computer environment. In the second stage, the sub-objectives of the study were determined. Fixed incorrectly invalidated values when parsing invalid values. While the missing values were separated, the assignment was made using the EM algorithm instead of the items left blank (Table 1).

**Table 1:** Skewness and Kurtosis values of variables.

Scale	N	Skewness	Kurtosis
Students’ undesirable behaviors	203	‑0.053	‑0.147
Strategy for preventing unwanted behaviors	203	‑0.480	‑0.314
Intervention methods for undesirable behaviors	203	0.134	‑0.334

According to Seçer (2015), evaluating the ‘skewness and kurtosis’ values is a more reliable method of evaluating the normal distribution assumption. Tabachnick and Fidell (2013) accept that normal distribution is achieved when the values of skewness and kurtosis are between ±1.50. As a result of the analyzes carried out, it was determined that the variables were within the stated ranges, that there were no extreme values and that they provided the normal distribution hypothesis, and the applicability of the parametric tests was reached.

## Results

The data of the study were analyzed in this section by applying the questions in the personal information form together with the statements in the scales of students’ undesirable behaviors, prevention strategy, and undesirable behavior intervention methods.

### Evaluation of demographic findings

In this part of the study, the personal characteristics of the teachers were examined, and descriptive analyses were made for their personal characteristics and presented in tables. According to the results of the frequency analysis, 66.0% of the participants included in the research are female and 34.0% are male. It was found that 42.9% of the participants were aged 41–50 and 36.0% were aged 31–40. To obtain more meaningful and accurate results, the group aged 60 and over was combined with the group aged 51–60 and renamed 51 years and over and they are the 21.1% of the population.

In terms of seniority, according to the frequency analysis results, 3.0% of the participants had less 5 years of seniority, 22.7% of the participants were between 21 and 25 years, 20.2% between 11 and 15 years, 18.2% between 6 and 10 years, 18.2% over 26 years, and 17% It is seen that 7 of them have professional experience between 16 and 20 years. In addition, to obtain more meaningful and accurate results, the 5 years and below group was combined with the 6–10 years group and renamed as 10 years and below.

About 24.1% of the participants are pre-school and classroom teachers, 21.7% are physical education/painting/music/technology design instructors, and 18.2% are mathematics and science teachers, according to the frequency analysis results. In addition, to acquire more meaningful and reliable findings, professional branches and foreign language groups were integrated (36.0%).

It was determined that 46.3% of the participants were secondary school teachers, 30.0% were high school teachers, 23.6% were primary school teachers, and 75.4% of the participants were undergraduate graduates. In addition, to obtain more meaningful and accurate results, the associate degree group was combined with the undergraduate group, and the educational status distribution of the participants was presented.

### Descriptive analysis of scales

Variables of the research are students’ undesirable behaviors, the strategy of preventing undesirable behaviors, and the methods of intervention to undesirable behaviors. This part of the research consists of descriptive statistics about the variables.

The total average of the answers given by the teachers involved in the study to the statements of students’ undesirable behaviors was found to be 37.38 ± 9.14, the minimum score was 15.00, and the maximum score was 60.00.

The total average of the answers given by the teachers involved in the study to the statements of the strategy of preventing undesirable behaviors was determined as 40.66 ± 5.88, the minimum score was 25.00, and the maximum score was 50.00.

It was observed that the total average of the answers given by the teachers involved in the study to the statements of intervention methods for undesirable behaviors was 32.12 ± 4.79, with a minimum score of 22.00 and a maximum score of 46.00.

### Difference tests

In this part of the study, the differences between the gender, age, professional experience, branch of the teachers constituting the research group, and the total scores of the students’ undesirable behaviors, the strategy of preventing undesirable behaviors, and the intervention methods for undesirable behaviors are discussed (Table 2).

**Table 2:** Differentiation of total scores obtained from variables by gender.

Variables	Gender	f	x̅	SD	t	p
Students’ undesirable behaviors	Male	69	35.29	9.79	‑2.374	0.019
	Female	134	38.47	8.63		
Intervention methods for undesirable behaviors	Male	69	39.19	5.88	‑2.593	0.10
	Female	134	41.42	5.76		
Strategy for preventing unwanted behaviors	Male	69	31.09	4.92	0.985	0.029
	Female	134	32.66	4.66		

According to the analysis result, the scores of teachers’ intervention methods for undesirable behaviors differ statistically according to their gender (*p* < 0.05) and male teachers’ scores for intervention methods against undesirable behaviors are higher than that of female teachers. In addition, there was no statistically significant difference in the scores of teachers’ students’ undesirable behaviors and strategies to prevent undesirable behaviors according to their gender (*p* > 0.05). In other words, while the gender of the teachers and the intervention methods for undesirable behaviors create a difference between the group total scores, the students’ undesirable behaviors and the strategy of preventing undesirable behaviors do not make a difference between the group total scores (Table 3).

**Table 3:** Difference status of total scores obtained from variables by age.

Variables	Age status	f	x̅	SD	F	p	Group difference
Students’ undesirable behaviors	(1) Between 20 and 30 years	15	42.33	6.20	4.997	0.002	1–3
	(1) Between 20 and 30 years	73	39.56	9.25			2–3
	(3) Between 41 and 50 years	87	35.31	9.29			
	(4) Age 51 and over	28	35.54	7.68			
Intervention methods for undesirable behaviors	(1) Between 20 and 30 years	15	43.60	5.25	2.659	0.049	1–4
	(2) Between 31 and 40 ages	73	41.14	6.07			
	(3) Between 41 and 50 years	87	40.41	5.73			
	(4) Age 51 and over	28	38.61	5.68			
Strategy for preventing unwanted behaviors	(1) Between 20 and 30 years	15	34.07	4.73	3.145	0.026	1–4
	(2) Between 31 and 40 ages	73	33.07	4.67			
	(3) Between 41 and 50 years	87	31.41	4.80			
	(4) Age 51 and over	28	30.86	4.59			

The results of the analysis displayed that teachers’ scores for students’ undesirable behaviors, intervention methods for undesirable behaviors, and strategies to prevent undesirable behaviors differ statistically according to their age (*p* < 0.05). On the other hand, it was determined that teachers aged 41–50 years had lower student misbehavior scores than teachers aged 20–30 and 31–40 years, which was statistically significant. In addition, it was determined that the scores of teachers aged 20–30 on intervention methods and strategies to prevent undesirable behaviors were higher than teachers aged 51 and over, and this situation was statistically significant. In other words, the age of the teachers, the undesirable behaviors of the students, the intervention methods for the undesirable behaviors, and the strategy of preventing the undesirable behaviors make a difference between the group total scores (Table 4).

**Table 4:** Differentiation status of total scores obtained from variables by occupational branch.

Variables	Professional branch	f	x̅	SD	F	p	Group difference
Students’ unwanted behaviors	(1) Pre-school and class teacher	49	36.14	8.28	2.489	0.033	2➔6
	(2) Physical education/painting/music/technology design	44	34.11	8.70			
	(3) Mathematics and science	37	38.62	10.56			
	(4) Turkish/TDE	21	38.52	9.14			
	(5) History/geography/social studies/religious culture/philosophy/guidance	30	39.50	8.47			
	(6) Professional branches and foreign language	22	40.68	8.68			
Intervention methods for undesirable behaviors	(1) Pre-school and class teacher	49	42.29	5.79	2.365	0.041	1➔5
	(2) Physical education/painting/music/technology design	44	40.14	6.05			
	(3) Mathematics and science	37	39.27	6.06			
	(4) Turkish/TDE	21	41.29	5.29			
	(5) History/geography/social studies/religious culture/philosophy/guidance	30	38.77	5.64			
	(6) Professional branches and foreign language	22	42.41	5.39			
Strategy for preventing unwanted behaviors	(1) Pre-school and class teacher	49	33.55	4.35	2.236	0.050	1➔2
	(2) Physical education/painting/music/technology design	44	30.52	5.18			
	(3) Mathematics and science	37	31.73	4.87			
	(4) Turkish/TDE	21	32.48	4.17			
	(5) History/geography/social studies/religious culture/philosophy/guidance	30	31.67	4.24			
	(6) Professional branches and foreign language	22	33.14	5.41			

According to the results of the analysis, teachers’ scores for students’ undesirable behaviors, intervention methods for undesirable behaviors, and strategies to prevent undesirable behaviors differ statistically significantly according to their branch (*p* < 0.05). On the other hand, professional branches and foreign language teachers have higher scores for students’ undesirable behaviors than physical education/painting/music/technology design teachers, and pre-school and classroom teachers’ scores for intervention methods for undesirable behaviors are history/geography/social studies/religious culture/philosophy/guidance. It was stated that the scores of the pre-school and classroom teachers on preventing unwanted behaviors were higher than the physical education/painting/music/technology design teachers, and this situation was statistically significant. In other words, the branch of the teachers, the undesirable behaviors of the students, the intervention methods for the undesirable behaviors, and the strategy of preventing the undesirable behaviors create a difference between the group total scores.

## Discussion, recommendations, and conclusion

When the personal characteristics of the teachers are examined; 66.0% of the teachers included in the research are female, 42.9% are 41–50 years old, 22.7% have 21–25 years of experience, 24.1% are pre-school, and classroom teachers, 46%, It was determined that 3 of them were secondary school teachers and 75.4% of them were only holding undergraduate degrees. When the differences between the gender, age, professional experience, branch, type of school, and education of the teachers constituting the research group and the total scores of the students’ undesirable behaviors, the strategy of preventing undesirable behaviors, and the intervention methods for undesirable behaviors are considered, the following are determined:

- While the professional experience of the teachers, the undesirable behaviors of the students, and the group total scores show a difference, the methods of intervention to the undesirable behaviors and the strategy of preventing undesirable behaviors did not show a difference between the group total scores explained.
- Teachers’ branch, students’ undesirable behaviors, intervention methods for undesirable behaviors, and strategy to prevent undesirable behaviors create differences between group total scores. The type of school where the teachers work and the undesired behaviors of the students do not show a difference between the group total scores, while the methods of intervention to the undesirable behavior and the strategy of preventing undesirable behaviors make a difference between the group total scores.

As a result, no difference was found between the group total scores of teachers’ education, students’ undesirable behaviors, intervention methods for undesirable behaviors, and strategy to prevent undesirable behaviors. Following sustainable suggestions can be made prior to the findings of the data. Such as to prevent unpredictable educational crises such as a pandemic, teachers should be trained on all components of the distance education system. East Asia countries are the ones that were able to benefit from lessons learned and to respond quickly to new crises of coronavirus (Joshi, 2021). In order to reach efficient lessons in distance education, it is important to provide teachers with access to fast and uninterrupted Internet connection.

It will be convenient for teachers to conduct lessons in the virtual classroom in a separate study room, if possible, which is not noisy, and where they will not be disturbed by anyone else. In order to ensure proper physical order for the students, it is important to cooperate with and guide the students’ parents. Similarly, Lassoued et al. (2020) states that one of the obstacles to achieving quality in distance learning during the COVID-19 pandemic was the weak Internet speed in many remote areas, and the consequent interruptions in broadcasting and the impediment to following lessons.

This study finds out that in a more effective and convenient evaluation environment, process-oriented evaluation approaches such as the digital product file that centers on the student can be used. Edumadze’s et al. (2022) findings are in parallel to suggest that teachers should instigate efforts to improve the students’ perception concerning the relevance of them-learning devices and technology adaptation in institutions of higher learning.

It can be concluded that regulating the behavior of students in virtual classrooms would be beneficial for teachers to create lists of rules appropriate for the nature of virtual classrooms. This would easily support classroom communication and students’ participation in the lessons and teachers would inevitably include applications such as chat, hand raise, small group activities, whiteboards in the structure of the course along with audio, webcam image, and screen sharing in these distant lectures.

## Data availability statement

The datasets presented in this study can be found in online repositories. The names of the repository/repositories and accession number(s) can be found in the article/supplementary material.

## Ethics statement

The studies involving human participants were reviewed and approved by Ethical Committee Board of Near East University. The patients/participants provided their written informed consent to participate in this study.

## Author contributions

ÖE prepared the ıntroduction and conceptualization of the artıcle. MB carried out the supervision of the article and helped to the generation of the methodology. All authors contributed to the article and approved the submitted version.

## Conflict of interest

The authors declare that the research was conducted in the absence of any commercial or financial relationships that could be construed as a potential conflict of interest.

## Publisher’s note

All claims expressed in this article are solely those of the authors and do not necessarily represent those of their affiliated organizations, or those of the publisher, the editors and the reviewers. Any product that may be evaluated in this article, or claim that may be made by its manufacturer, is not guaranteed or endorsed by the publisher.
